# Supplementary material for: Assembly-active and -inactive forms of HBV capsid protein provide distinctly different binding sites for capsid assembly modulators
Source: PLoS Pathog. 2026 Oct 9;22(10):e1014700. doi: 10.1371/journal.ppat.1014700 (PMC13705612; doi:10.1371/journal.ppat.1014700)
Supplement: Supplementary file 1 [file ppat.1014700.s001.docx]

**Appendix:**

**Assembly-active and -inactive forms of HBV capsid protein provide distinctly different binding sites
for capsid assembly modulators**

Liam W. Scott,^1^ Carolina Pérez-Segura,^2^ Jodi A. Hadden-Perilla,^2^ Adam Zlotnick^1^*

1. Molecular and Cellular Biochemistry Department, Indiana University, Bloomington, IN 47405 United States
2. Department of Chemistry & Biochemistry, University of Delaware, Newark, Delaware 19716, United States

*	Corresponding Author:

Adam Zlotnick

Molecular and Cellular Biochemistry Department

Indiana University, Bloomington, IN 47405 United States

azlotnic@iu.edu

**Table A: List of HBV-Cp structures used in study.** 2G33 is used as “apo Cp150” in all studies and 4BMG is used as “apo Cp149-Y132A” in all studies. The only other CAM bound Cp149-Y132A structure that we found was 8W9G. 8W9G does not have an associated publication as of this time, and so it was not included in this study. 7OD4 was used as the alignment structure for **Fig 2** and **Appendix Fig B**. 3J2V A/A pocket was used to align data for **Fig 3-4**. 1QGT was used to align pockets for **Appendix Fig C**. 3J2V was utilized as an apo form of Cp183 to act as a reference to compare against in **Fig 6**, **Appendix Fig B**, and **Appendix Fig D**.

* Structures not used in this study: 3KXS crystallized with a different packaging arrangement from 4BMG and the other CAM bound Cp149-Y132A structures. 6J10 has a different chain naming nomenclature from all other Cp149-Y132A structures.

**Figure B: Hexameric units from capsids and Cp149-Y132A sheets have different curvatures.** External/Top view (left) and side view (right) of an alignment of apo and CAM bound hexameric units from apo Cp183 (PDB:3J2V), apo Cp149 (PDB:1QGT), Cp150 (apo PDB:2G33, + CAM PDB:2G34), and Cp149-Y132A (apo PDB:4BMG, + CAM PDB:5WRE). Only the D chain (gray) was aligned using ChimeraX’s Matchmaker function. This alignment shows that hexamers of capsids have greater curvature than hexamers from Cp149-Y132A sheets, independent of CAM binding.

**Figure C: Aligned pocket alpha-carbons are highly similar across apo and CAM bound structures of Cp150 and Cp149-Y132A.** (**a**) Alignment of residues 100-120 and 23-37 of Linvencorvir bound Cp149-Y132A (PDBID:8I71) and Hap-18 bound Cp150 (PDBID:5D7Y) to the B pocket of RNA filled, apo Cp183 (not shown, PDBID: 7OD4). (**b**) C-alpha to C-alpha distance of the aligned B pockets of several CAM bound Cp149-Y132A and Cp150 structures from the aligned-to apo Cp183 structure. Overall, samples do not vary more than 1.0 Å from the aligned-to pocket until approaching the portion of helix5 that acts as the cap for the neighboring pocket. (**Appendix Table A**)

**Figure D: Aligned CAM site shapes are similar in Cp149-Y132A structures and variable in capsid structures.** Prior to calculating CAM site shape and volume using VMD’s *Measure volinterior* with fuzzy boundary conditions, all structures were aligned to the B pocket of Cp149 (PDBID:1QGT) using residues 100-120 and 23-37. This allows for overlays of the CAM sites from different quasi-equivalent sites. Stick structures of a CAM displayed at top, an image of that CAM bound in the B site of its Cp150 or Cp149-Y132A structure with the B pocket displayed as a gray surface in the middle, and that same B pocket with shown with an overlay of all VMD calculated quasi-equivalent site shapes from that structure. We note that CAM site volume shapes are nearly identical across overlaid Cp149-Y132A quasi-equivalent sites, while Cp150 site volume shapes show variability in shape and orientation between quasi-equivalent sites. (**Appendix Table A**)

**Figure E: CAM interacting residues: Cp150.** Residues with a 0.5 Angstrom VDW overlap with bound CAMs in Cp150 HBc structures. Purple = interacting, cyan = not interacting. (**Appendix Table A**)

**Figure F: CAM interacting residues: Cp149-Y132A.** Residues with a 0.5 Angstrom VDW overlap with bound CAMs in Cp149-Y132A HBc structures. Purple = interacting, cyan = not interacting. (**Appendix Table A**)

**Figure G: Residues that form hydrogen bonds with CAMs: Cp149-Y132A.** Residues that form hydrogen bonds with bound CAMs in Cp149-Y132A HBc structures. For each structure, the CAM formed hydrogen bonds with specified residues in all 6 available quasi-equivalent CAM sites. Purple = interacting, cyan = not interacting. (**Appendix Table A**)

**Figure H: Residues that form hydrogen bonds with CAMs: Cp150.** Residues that form hydrogen bonds with bound CAMs in Cp150 HBV-Cp structures. Purple = interacting, cyan = not interacting. Blank columns represent quasi-equivalent sites that are not CAM bound. (**Appendix Table A**)
